# RETRACTION: Involvement of Heme Oxygenase‐1 Induction in the Cytoprotective and Immunomodulatory Activities of *Viola patrinii* in Murine Hippocampal and Microglia Cells

**DOI:** 10.1155/ecam/9825416

**Published:** 2026-02-10

**Authors:** Evidence-Based Complementary and Alternative Medicine

RETRACTION: B. Li, D‐S. Lee, H‐G. Choi, K‐S. Kim, G‐S. Jeong, R. B. An, and Y‐C. Kim, “Involvement of Heme Oxygenase‐1 Induction in the Cytoprotective and Immunomodulatory Activities of *Viola patrinii* in Murine Hippocampal and Microglia Cells,” *Evidence-Based Complementary and Alternative Medicine*, vol. 2012 (2012). https://doi.org/10.1155/2012/128019.

The above article, published online on 24 March 2012 in Wiley Online Library (https://wileyonlinelibrary.com), has been retracted by John Wiley & Sons Ltd.

The retraction has been agreed following an investigation undertaken by the publisher, which identified multiple instances of inappropriately overlapping figures as follows:

- In Figure 3a, the Western blot bands corresponding to actin expression share an unexpected similarity with the actin bands in Figure 5b of [1], which were obtained under different experimental conditions.
- In Figure 5c, the Western blot bands corresponding to actin expression appear similar to the p‐38 bands in Figure 9a of [2].
- Both sets of the Western Blot bands in Figure 6a share similarity with the bands in Figure 3c of [1]. Although the bands denote the expression of the same proteins, the experimental conditions are different.
- Multiple bands from Figure 9b share unexpected similarities with Western Blot results in multiple publications by the author group, where they are used to represent different markers [1, 3, 4].

As a result of our investigation, the conclusions of the article can no longer be considered reliable, and it is therefore retracted.

The authors disagree with this retraction.
